# The pathological role of vascular aging in cardio-metabolic disorder

**DOI:** 10.1186/s41232-016-0021-6

**Published:** 2016-08-16

**Authors:** Goro Katsuumi, Ippei Shimizu, Yohko Yoshida, Tohru Minamino

**Affiliations:** 1 Department of Cardiovascular Biology and Medicine, Niigata University Graduate School of Medical and Dental Sciences, 1-757 Asahimachidori, Chuo-ku, Niigata, 951-8510 Japan; 2 Division of Molecular Aging and Cell Biology, Niigata University Graduate School of Medical and Dental Sciences, 1-757 Asahimachidori, Chuo-ku, Niigata, 951-8510 Japan

**Keywords:** p53, Cellular senescence, Endothelial cell, Heart failure, Diabetes

## Abstract

Chronological aging is linked to cellular senescence, and there is accumulating evidence for a pathological role of cellular senescence in age-related disorders such as obesity, diabetes, and heart failure. The protein p53 has a central role in cellular senescence, and p53 expression in cardiomyocytes, vascular endothelial cells, adipocytes, and immune cells leads to the development of heart failure and diabetes. It is widely accepted that formation of capillary networks is critical for morphogenesis of organs and maintenance of homeostasis. Capillary rarefaction and hypoxia promote pathological changes in the myocardium of the failing heart, causing systolic dysfunction. Capillary rarefaction and hypoxia also cause dysfunction of brown adipose tissue (BAT), leading to systemic metabolic disorders with promotion of diabetes. Vascular endothelial cell senescence develops in heart failure and diabetes and is responsible for progression of these age-related disorders. In a murine model of left ventricular pressure overload, increased expression of p53 in vascular endothelial cells and bone marrow cells promotes inflammatory cell infiltration into the heart, contributing to cardiac remodeling and systolic dysfunction. Metabolic stress up-regulates p53 expression in endothelial cells, while reducing the phosphorylation of endothelial nitric oxide synthase (eNOS) and glucose transporter (GLUT)1 expression in these cells. These changes lead to suppression of mitochondrial biogenesis and glucose uptake in the skeletal muscle and promote the development of systemic metabolic dysfunction. Suppression of vascular aging and vascular dysfunction is critically important for maintenance of organ homeostasis and is essential for prevention or treatment of heart failure, obesity, and diabetes.

## Background

Chronological aging increases the risk of age-related disorders such as obesity, diabetes, and heart failure. Aging also occurs at the cellular level. Shortening of telomeres associated with cell division triggers the DNA damage response and cellular senescence, which is known as replicative senescence and is mainly mediated via a p53 signaling pathway [10, 12]. In addition, stress due to factors such as cytokines and reactive oxygen species also leads to p53-mediated cellular senescence that is termed premature senescence [27]. Senescent cells are characterized by growth arrest associated with various alterations of gene expression [10, 24, 32, 37].

Obesity and its associated disorders are among the top health problems in many societies. Obesity promotes pathological processes that contribute to atherosclerotic disease, heart failure, and diabetes, and there is evidence for a causal role of cellular senescence in these diseases. Metabolic stress leads to p53-induced cellular senescence in adipocytes, resulting in adipose tissue inflammation and systemic metabolic dysfunction [16]. Obesity has also been linked to elevated p53 expression in vascular endothelial cells, which reduces the activation of endothelial nitric oxide synthase (eNOS) and glucose transporter (GLUT)1 in these cells and contributes to suppression of mitochondrial biogenesis and glucose uptake in the skeletal muscle, leading to systemic metabolic dysfunction [41]. In addition, capillary rarefaction and hypoxia cause dysfunction of brown adipose tissue (BAT) and promote systemic metabolic abnormalities [28]. Recently, vascular senescence was shown to have a pathological role in the progression of heart failure. In a murine model of left ventricular pressure overload, increased adrenergic signaling associated with heart failure was found to up-regulate p53 expression in both endothelial cells and bone marrow cells, contributing to cardiac inflammation and remodeling [42]. Heart failure also promotes p53-induced adipocyte senescence and visceral fat inflammation, leading to the development of systemic insulin resistance and hyperinsulinemia. In turn, hyperinsulinemia contributes to cardiac hypertrophy via the Akt signaling pathway, possibly because cardiac tissue is slow to develop insulin resistance, and activation of this pathway has a detrimental effect on cardiac homeostasis by promoting pathological cardiomyocyte hypertrophy, cardiomyocyte/capillary mismatch, and hypoxia [29].

It is widely accepted that formation of vascular networks is critically important for organ morphogenesis and for maintenance of homeostasis, while cellular senescence has a pathological role in promoting vascular dysfunction that leads to organ dysfunction and systemic metabolic disorders [6, 28, 41, 42]. In this review, we discuss the role of vascular aging in cardio-metabolic disorders.

## Pathological role of vascular aging/dysfunction in heart failure

**Fig. 1:**
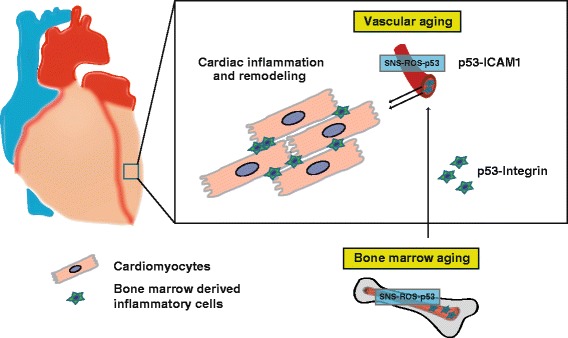
The role of vascular and bone marrow aging in heart failure. Activation of sympathetic nervous system (SNS)/ROS/p53 signaling promotes the interaction between endothelial cells and bone marrow-derived inflammatory cells by the up-regulation of ICAM-1 and integrin expression to exacerbate cardiac dysfunction

The prognosis of severe heart failure is still very poor, and it is urgent to find new therapeutic targets for this disorder [2]. Hypertensive heart disease is among the chief causes of heart failure. When cardiac tissue is exposed to pressure overload, cardiac hypertrophy occurs in step with angiogenesis as an adaptive response to maintain systolic function. Sustained pressure overload promotes the transition to decompensated heart failure, which features excessive cardiomyocyte hypertrophy uncoordinated with the angiogenic response [33]. The resulting capillary rarefaction and hypoxia contribute to cardiac remodeling and systolic dysfunction. It was reported that increased cardiac expression of p53, which induces cellular senescence, promotes cardiac dysfunction in a murine model of pressure overload. In addition, p53 suppresses cardiac angiogenesis via inhibition of hypoxia-inducible factor 1α (Hif-1α) and vascular endothelial growth factor (VEGF), thus promoting cardiac hypoxia and remodeling [25]. It is well accepted that sterile inflammation contributes to the progression of cardiac remodeling associated with heart failure, but the mechanistic link between p53 and inflammation in the failing heart has been unclear [7]. Recently, activation of p53 signaling in vascular endothelial cells and bone marrow cells was reported to be the underlying cause of cardiac inflammation in a murine model of left ventricular (LV) pressure overload [42]. In this model, p53 expression was significantly increased in cardiac microvascular endothelial cells and bone marrow cells, leading to up-regulation of intercellular adhesion molecule (ICAM)-1 expression by endothelial cells and an increase of integrin alpha-L in macrophages. Genetic deletion of p53 in endothelial cells or bone marrow cells reduced the expression of these adhesion molecules, suppressed inflammatory cell infiltration into cardiac tissue, inhibited production of pro-inflammatory cytokines, and ameliorated cardiac dysfunction due to LV pressure overload. Conversely, forced expression of p53 in bone marrow cells led to exacerbation of cardiac inflammation and systolic dysfunction. It is well known that activation of the sympathetic nervous system (SNS) occurs in heart failure patients and is associated with a poor prognosis [3]. In the LV pressure overload model, norepinephrine markedly increased the level of reactive oxygen species (ROS) and p53 expression in macrophages and endothelial cells, while inhibition of adrenergic signaling through suppression of beta-2 adrenergic receptor expression in endothelial cells or bone marrow cells decreased ROS and p53 levels, and also ameliorated cardiac inflammation and systolic dysfunction due to pressure overload. These results suggest that activation of SNS/ROS/p53 signaling promotes interaction between endothelial cells and bone marrow-derived inflammatory cells via up-regulation of ICAM-1 and integrin expression, resulting in exacerbation of cardiac dysfunction [42] (Fig. 1). It was also reported that increased p53 signaling in endothelial cells led to capillary rarefaction in cardiac tissue in a murine model of LV pressure overload, while depletion of endothelial cell p53 ameliorated capillary rarefaction, improved cardiac function, and suppressed cardiac fibrosis/remodeling [8]. Cardiac expression of p53 is up-regulated by LV pressure overload, but this change is significantly suppressed by endothelial cell p53 depletion. These results indicate that infiltration of inflammatory cells into cardiac tissue (promoted by endothelial cell senescence) is the initial step in the process of cardiac remodeling due to LV pressure overload, and this process is accelerated by capillary rarefaction and hypoxia [8, 25, 42]. It is well accepted that the non-selective β-blocker, carvedilol, has a cardioprotective effect contributing for the better clinical outcomes in severe heart failure patients with reduced ejection fraction. Carvedilol is known to bind preferentially to beta-2 adrenergic receptor, and it may exert its biological effects via the suppression of SNS/ROS/p53 signaling mediated via beta-2 adrenergic receptor in endothelial cells. Accordingly, suppression of endothelial cell senescence could be a new therapeutic target for heart failure associated with reduced cardiac systolic function.

It is well known that approximately half of the heart failure is classified as heart failure with preserved ejection fraction (HFpEF). HFpEF is known to be predominant among elderly individuals. Overweight/obesity, hypertension, and diabetes mellitus are also known to become the risk factors for promoting this pathological condition. Cardiomyocyte hypertrophy and interstitial fibrosis, associated with incomplete relaxation of myocardial strips and cardiomyocyte stiffness, develop in HFpEF [13, 26, 38]. Recently, coronary microvascular inflammation is thought crucial for the development of HFpEF [22]. Considering that cellular senescence has a central role in inducing vascular dysfunction, it is highly possible that this would promote pathologies in HFpEF. Further studies are needed to analyze the role of vascular aging in this critical disorder.

## Pathological role of vascular aging/dysfunction in obesity

### Skeletal muscle

**Fig. 2:**
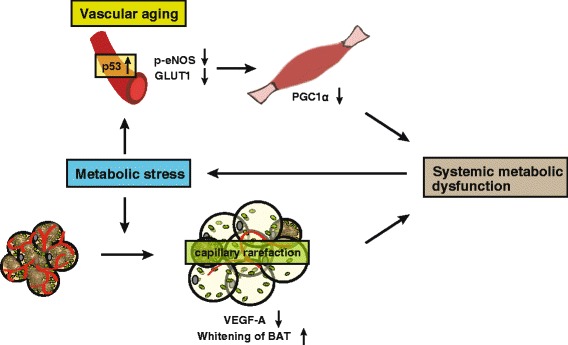
The role of vascular aging and hypoxia-induced BAT dysfunction in obesity. Metabolic stress up-regulates p53 in endothelial cells and reduces the activation of eNOS and GLUT1 level. This leads to reduced PGC1-α expression in skeletal muscle leading to the development of systemic metabolic dysfunction. In brown adipose tissue (BAT), metabolic stress promotes capillary rarefaction by the suppression of VEGF-A, and this leads to the whitening of BAT

The skeletal muscle makes a major contribution to glucose disposal, so maintenance of skeletal muscle homeostasis is crucial for metabolic health. Metabolic stress induces accumulation of lipids and causes inflammation that promotes insulin resistance in the skeletal muscle, contributing to the development of systemic insulin resistance [15, 23]. Capillaries have an important role in the regulation of skeletal muscle metabolism. An increase of p53 in the vascular endothelium was reported in a murine model of dietary obesity [41]. In this model, genetic depletion of endothelial p53 reduced the accumulation of visceral and subcutaneous fat and led to improvement of systemic insulin resistance. eNOS up-regulates peroxisome proliferator-activated receptor-γ coactivator-1α (PGC1-α) in the skeletal muscle, while p53 suppresses the activation of eNOS. It was found that the depletion of endothelial cell p53 promoted glucose uptake by the skeletal muscle via up-regulation of GLUT1 expression in endothelial cells. These findings suggest that suppression of vascular aging contributes to better metabolic health by promoting mitochondrial biogenesis in the skeletal muscle [41] (Fig. 2).

### Brown adipose tissue

Several types of fat exist in the body, including white, brown, and beige adipose tissue. BAT was initially characterized as a thermogenic organ that is abundantly expressed in newborn infants and small rodents, but subsequent studies have shown that adult humans also have BAT [4]. In addition to its role in thermogenesis, BAT is now known to make a large contribution to the regulation of systemic metabolism [1, 18, 34]. In adults, BAT activity was found to decline with obesity and aging, but the detailed mechanisms involved were uncertain. BAT is highly vascular, and metabolic stress was recently shown to suppress angiogenesis by reducing the expression of vascular endothelial growth factor-A (VEGF-A), a major pro-angiogenic molecule, in brown adipocytes due to accumulation of fatty acids. This leads to capillary rarefaction and hypoxia, which affect BAT much more prominently than white adipose tissue (WAT), leading to “whitening” of BAT that is associated with diminished β-adrenergic signaling, accumulation of large lipid droplets, and mitochondrial dysfunction or loss. These changes of the BAT microenvironment impair thermogenesis and promote systemic metabolic dysfunction. Whitening of BAT has also been observed after ablation of *Vegfa* in the adipose tissue of non-obese mice, which demonstrated impairment of systemic glucose metabolism and reduction of thermogenesis. Importantly, specific introduction of *Vegfa* into the BAT of mice with dietary obesity restored its vascularity and ameliorated brown adipocyte dysfunction, and this re-browning of BAT was associated with improvement of insulin sensitivity. These data indicate that overnutrition promotes hypoxia in BAT, causing it to whiten through mitochondrial dysfunction and loss, and subsequently contributing to impaired systemic glucose metabolism [28] (Fig. 2). Recently, the expression of an anti-angiogenic VEGF-A splice isoform (VEGF-A165b) was shown to increase upon metabolic stress and inhibit vascularization in ischemic hind limbs [14]. The role of VEGF-A165b in the maintenance of BAT homeostasis remains an open question.

### White adipose tissue

WAT was initially thought to be mainly involved in energy storage, but it is now well known to be an active endocrine organ secreting humoral mediators called “adipokines” [20]. Metabolic stress induces the influx of fatty acids into white adipocytes, leading to increased production of inflammatory cytokines and promoting systemic metabolic dysfunction. Angiogenic factors have been reported to have a critical role in the maintenance of homeostasis in WAT [5, 35, 36]. In obese patients and obese mice, WAT displays capillary rarefaction and hypoxia associated with the infiltration of macrophages and increased production of pro-inflammatory cytokines. In obesity, the VEGF-A level has variously been reported to be increased [11, 40], decreased [9, 17, 21], or unchanged [39], but there is a consensus that inadequate angiogenesis occurs during WAT expansion associated with metabolic stress. Disruption of VEGF expression in the fat cells of mice leads to capillary rarefaction and hypoxia in visceral WAT along with increased *Tnf* expression, and the mice develop systemic metabolic dysfunction when fed a high-calorie diet. In contrast, forced VEGF expression in fat cells increases the vascularity of adipose tissue and ameliorates systemic metabolic dysfunction in mice with dietary obesity. These findings indicate that capillary network formation has a crucial role in WAT homeostasis and metabolic health [5, 35, 36].

Recently, impaired angiogenesis in visceral adipose tissue was reported to associate with high VEGF-A165b expression in the fat, suggesting that the inhibition of VEGF-A165b would have a therapeutic potential to maintain homeostasis in visceral fat, contributing to the suppression of systemic metabolic dysfunction [19].

In both white and brown adipose tissue, molecules or mechanisms contributing to the up-regulation of angiogenic VEGF are yet to be defined. Studies indicate that hypoxia-inducible factor 1α (HIF1α) in the visceral fat is increased in response to obesity. In cardiac tissue and other organs, HIF1α is well accepted as a critical regulator for angiogenesis [25]. Interestingly, in both fats, HIF1α does not induce angiogenesis; instead, it activates fibrotic response or autophagy and disturbs homeostasis in these organs [9, 28]. Further studies are needed to explore mechanisms that would promote angiogenesis in white and brown adipose tissue.

## Conclusions

Capillaries are critically involved in maintenance of homeostasis in the heart, BAT and WAT, and skeletal muscle [5, 25, 28, 35, 36, 41, 42]. Studies have shown that endothelial cell senescence leads to cardiac inflammation, capillary rarefaction, and hypoxia, thereby promoting pathologic cardiac remodeling in response to LV pressure overload [25, 42]. In a murine model of LV pressure overload, the downregulation of endothelial cell expression of p53 (a critical regulator of cellular senescence) has been shown to inhibit cardiac inflammation, promote angiogenesis, and protect cardiac function [42]. Studies performed in murine models of obesity have shown that capillary rarefaction also develops in BAT as a response to metabolic stress. Accumulation of lipids leads to the suppression of VEGF-A, a critical regulator of angiogenesis, and induces capillary rarefaction and hypoxia, promoting the whitening of brown fat and systemic metabolic dysfunction. Conversely, up-regulation of *Vegfa* expression promotes the re-browning of whitened BAT and improves systemic metabolic health [28]. Endothelial cell senescence is linked with metabolic dysfunction in skeletal muscle via suppression of mitochondrial biogenesis and contributes to systemic metabolic dysfunction [41]. These results provide evidence that maintenance of vascular homeostasis through regulation of endothelial cell senescence is critically important for suppressing pathologic changes associated with heart failure, obesity, and diabetes. It has been reported that systemic insulin resistance develops during heart failure in humans and mice. Excessive lipolysis in visceral fat promotes adipose tissue inflammation during LV pressure overload and leads to systemic insulin resistance with hyperinsulinemia [30], while excessive insulin signaling has been reported to induce pathological cardiac hypertrophy associated with capillary rarefaction and hypoxia [29]. Studies have shown that p53-induced senescence of adipocytes causes the development of adipose tissue inflammation and systemic insulin resistance in animal models of obesity or heart failure, contributing to the progression of these age-related disorders [16, 30, 31]. Recent studies have also indicated that endothelial cell senescence has a pathological role in systemic insulin resistance through impairment of skeletal muscle metabolism [41]. Thus, maintenance of vascular homeostasis is essential in the management of obesity, diabetes, and heart failure.
